# Systematic review or scoping review? Guidance for authors when choosing between a systematic or scoping review approach

**DOI:** 10.1186/s12874-018-0611-x

**Published:** 2018-11-19

**Authors:** Zachary Munn, Micah D. J. Peters, Cindy Stern, Catalin Tufanaru, Alexa McArthur, Edoardo Aromataris

**Affiliations:** The Joanna Briggs Institute, The University of Adelaide, 55 King William Road, North Adelaide, 5005 South Australia

**Keywords:** Systematic review, Scoping review, Evidence-based healthcare

## Abstract

**Background:**

Scoping reviews are a relatively new approach to evidence synthesis and currently there exists little guidance regarding the decision to choose between a systematic review or scoping review approach when synthesising evidence. The purpose of this article is to clearly describe the differences in indications between scoping reviews and systematic reviews and to provide guidance for when a scoping review is (and is not) appropriate.

**Results:**

Researchers may conduct scoping reviews instead of systematic reviews where the purpose of the review is to identify knowledge gaps, scope a body of literature, clarify concepts or to investigate research conduct. While useful in their own right, scoping reviews may also be helpful precursors to systematic reviews and can be used to confirm the relevance of inclusion criteria and potential questions.

**Conclusions:**

Scoping reviews are a useful tool in the ever increasing arsenal of evidence synthesis approaches. Although conducted for different purposes compared to systematic reviews, scoping reviews still require rigorous and transparent methods in their conduct to ensure that the results are trustworthy. Our hope is that with clear guidance available regarding whether to conduct a scoping review or a systematic review, there will be less scoping reviews being performed for inappropriate indications better served by a systematic review, and vice-versa.

## Background

Systematic reviews in healthcare began to appear in publication in the 1970s and 1980s [1, 2]. With the emergence of groups such as Cochrane and the Joanna Briggs Institute (JBI) in the 1990s [3], reviews have exploded in popularity both in terms of the number conducted [1], and their uptake to inform policy and practice. Today, systematic reviews are conducted for a wide range of purposes across diverse fields of inquiry, different evidence types and for different questions [4]. More recently, the field of evidence synthesis has seen the emergence of scoping reviews, which are similar to systematic reviews in that they follow a structured process, however they are performed for different reasons and have some key methodological differences [5–8]. Scoping reviews are now seen as a valid approach in those circumstances where systematic reviews are unable to meet the necessary objectives or requirements of knowledge users. There now exists clear guidance regarding the definition of scoping reviews, how to conduct scoping reviews and the steps involved in the scoping review process [6, 8]. However, the guidance regarding the key indications or reasons why reviewers may choose to follow a scoping review approach is not as straightforward, with scoping reviews often conducted for purposes that do not align with the original indications as proposed by Arksey and O’Malley [5–10]. As editors and peer reviewers for various journals we have noticed that there is inconsistency and confusion regarding the indications for scoping reviews and a lack of clarity for authors regarding when a scoping review should be performed as opposed to a systematic review. The purpose of this article is to provide practical guidance for reviewers on when to perform a systematic review or a scoping review, supported with some key examples.

## Indications for systematic reviews

Systematic reviews can be broadly defined as a type of research synthesis that are conducted by review groups with specialized skills, who set out to identify and retrieve international evidence that is relevant to a particular question or questions and to appraise and synthesize the results of this search to inform practice, policy and in some cases, further research [11–13]. According to the Cochrane handbook, a systematic review ‘uses explicit, systematic methods that are selected with a view to minimizing bias, thus providing more reliable findings from which conclusions can be drawn and decisions made.’ [14] Systematic reviews follow a structured and pre-defined process that requires rigorous methods to ensure that the results are both reliable and meaningful to end users. These reviews may be considered the pillar of evidence-based healthcare [15] and are widely used to inform the development of trustworthy clinical guidelines [11, 16, 17].

A systematic review may be undertaken to confirm or refute whether or not current practice is based on relevant evidence, to establish the quality of that evidence, and to address any uncertainty or variation in practice that may be occurring. Such variations in practice may be due to conflicting evidence and undertaking a systematic review should (hopefully) resolve such conflicts. Conducting a systematic review may also identify gaps, deficiencies, and trends in the current evidence and can help underpin and inform future research in the area. Systematic reviews can be used to produce statements to guide clinical decision-making, the delivery of care, as well as policy development [12]. Broadly, indications for systematic reviews are as follows [4]:

1. Uncover the international evidence
2. Confirm current practice/ address any variation/ identify new practices
3. Identify and inform areas for future research
4. Identify and investigate conflicting results
5. Produce statements to guide decision-making

Despite the utility of systematic reviews to address the above indications, there are cases where systematic reviews are unable to meet the necessary objectives or requirements of knowledge users or where a methodologically robust and structured preliminary searching and scoping activity may be useful to inform the conduct of the systematic reviews. As such, scoping reviews (which are also sometimes called scoping exercises/scoping studies) [8] have emerged as a valid approach with rather different indications to those for systematic reviews. It is important to note here that other approaches to evidence synthesis have also emerged, including realist reviews, mixed methods reviews, concept analyses and others [4, 18–20]. This article focuses specifically on the choice between a systematic review or scoping review approach.

## Indications for scoping reviews

True to their name, scoping reviews are an ideal tool to determine the scope or coverage of a body of literature on a given topic and give clear indication of the volume of literature and studies available as well as an overview (broad or detailed) of its focus. Scoping reviews are useful for examining emerging evidence when it is still unclear what other, more specific questions can be posed and valuably addressed by a more precise systematic review [21]. They can report on the types of evidence that address and inform practice in the field and the way the research has been conducted.

The general purpose for conducting scoping reviews is to identify and map the available evidence [5, 22]. Arskey and O’Malley, authors of the seminal paper describing a framework for scoping reviews, provided four specific reasons why a scoping review may be conducted [5–7, 22]. Soon after, Levac, Colquhoun and O’Brien further clarified and extended this original framework [7]. These authors acknowledged that at the time, there was no universally recognized definition of scoping reviews nor a commonly acknowledged purpose or indication for conducting them. In 2015, a methodological working group of the JBI produced formal guidance for conducting scoping reviews [6]. However, we have not previously addressed and expanded upon the indications for scoping reviews. Below, we build upon previously described indications and suggest the following purposes for conducting a scoping review:

- To identify the types of available evidence in a given field
- To clarify key concepts/ definitions in the literature
- To examine how research is conducted on a certain topic or field
- To identify key characteristics or factors related to a concept
- As a precursor to a systematic review
- To identify and analyse knowledge gaps

## Deciding between a systematic review and a scoping review approach

Authors deciding between the systematic review or scoping review approach should carefully consider the indications discussed above for each synthesis type and determine exactly what question they are asking and what purpose they are trying to achieve with their review. We propose that the most important consideration is whether or not the authors wish to use the results of their review to answer a clinically meaningful question or provide evidence to inform practice. If the authors have a question addressing the feasibility, appropriateness, meaningfulness or effectiveness of a certain treatment or practice, then a systematic review is likely the most valid approach [11, 23]. However, authors do not always wish to ask such single or precise questions, and may be more interested in the identification of certain characteristics/concepts in papers or studies, and in the mapping, reporting or discussion of these characteristics/concepts. In these cases, a scoping review is the better choice.

As scoping reviews do not aim to produce a critically appraised and synthesised result/answer to a particular question, and rather aim to provide an overview or map of the evidence. Due to this, an assessment of methodological limitations or risk of bias of the evidence included within a scoping review is generally not performed (unless there is a specific requirement due to the nature of the scoping review aim) [6]. Given this assessment of bias is not conducted, the implications for practice (from a clinical or policy making point of view) that arise from a scoping review are quite different compared to those of a systematic review. In some cases, there may be no need or impetus to make implications for practice and if there is a need to do so, these implications may be significantly limited in terms of providing concrete guidance from a clinical or policy making point of view. Conversely, when we compare this to systematic reviews, the provision of implications for practice is a key feature of systematic reviews and is recommended in reporting guidelines for systematic reviews [13].

## Exemplars for different scoping review indications

In the following section, we elaborate on each of the indications listed for scoping reviews and provide a number of examples for authors considering a scoping review approach.

### To identify the types of available evidence in a given field

Scoping reviews that seek to identify the types of evidence in a given field share similarities with evidence mapping activities as explained by Bragge and colleagues in a paper on conducting scoping research in broad topic areas [24]. Chambers and colleagues [25] conducted a scoping review in order to identify current knowledge translation resources (and any evaluations of them) that use, adapt and present findings from systematic reviews to suit the needs of policy makers. Following a comprehensive search across a range of databases, organizational websites and conference abstract repositories based upon predetermined inclusion criteria, the authors identified 20 knowledge translation resources which they classified into three different types (overviews, summaries and policy briefs) as well as seven published and unpublished evaluations. The authors concluded that evidence synthesists produce a range of resources to assist policy makers to transfer and utilize the findings of systematic reviews and that focussed summaries are the most common. Similarly, a scoping review was conducted by Challen and colleagues [26] in order to determine the types of available evidence identifying the source and quality of publications and grey literature for emergency planning. A comprehensive set of databases and websites were investigated and 1603 relevant sources of evidence were identified mainly addressing emergency planning and response with fewer sources concerned with hazard analysis, mitigation and capability assessment. Based on the results of the review, the authors concluded that while there is a large body of evidence in the field, issues with its generalizability and validity are as yet largely unknown and that the exact type and form of evidence that would be valuable to knowledge users in the field is not yet understood.

### To clarify key concepts/definitions in the literature

Scoping reviews are often performed to examine and clarify definitions that are used in the literature. A scoping review by Schaink and colleagues^27^ was performed to investigate how the notion of “patient complexity” had been defined, classified, and understood in the existing literature. A systematic search of healthcare databases was conducted. Articles were assessed to determine whether they met the inclusion criteria and the findings of included articles were grouped into five health dimensions. An overview of how complexity has been described was presented, including the varying definitions and interpretations of the term. The results of the scoping review enabled the authors to then develop a complexity framework or model to assist in defining and understanding patient complexity [27].

Hines et al. [28] provide a further example where a scoping review has been conducted to define a concept, in this case the condition bronchopulmonary dysplasia. The authors revealed significant variation in how the condition was defined across the literature, prompting the authors to call for a ‘comprehensive and evidence-based definition’. [28]

### To examine how research is conducted on a certain topic

Scoping reviews can be useful tools to investigate the design and conduct of research on a particular topic. A scoping review by Callary and colleagues^29^ investigated the methodological design of studies assessing wear of a certain type of hip replacement (highly crosslinked polyethylene acetabular components) [29]. The aim of the scoping review was to survey the literature to determine how data pertinent to the measurement of hip replacement wear had been reported in primary studies and whether the methods were similar enough to allow for comparison across studies. The scoping review revealed that the methods to assess wear (radiostereometric analysis) varied significantly with many different approaches being employed amongst the investigators. The results of the scoping review led to the authors recommending enhanced standardization in measurements and methods for future research in this field [29].

There are other examples of scoping reviews investigating research methodology, with perhaps the most pertinent examples being two recent scoping reviews of scoping review methods [9, 10]. Both of these scoping reviews investigated how scoping reviews had been reported and conducted, with both advocating for a need for clear guidance to improve standardization of methods [9, 10]. Similarly, a scoping review investigating methodology was conducted by Tricco and colleagues^30^ on rapid review methods that have been evaluated, compared, used or described in the literature. A variety of rapid review approaches were identified with many instances of poor reporting identified. The authors called for prospective studies to compare results presented by rapid reviews versus systematic reviews.

### To identify key characteristics or factors related to a concept

Scoping reviews can be conducted to identify and examine characteristics or factors related to a particular concept. Harfield and colleagues (2015) conducted a scoping review to identify the characteristics of indigenous primary healthcare service delivery models [30–32]. A systematic search was conducted, followed by screening and study selection. Once relevant studies had been identified, a process of data extraction commenced to extract characteristics referred to in the included papers. Over 1000 findings were eventually grouped into eight key factors (accessible health services, community participation, culturally appropriate and skilled workforce, culture, continuous quality improvement, flexible approaches to care, holistic health care, self-determination and empowerment). The results of this scoping review have been able to inform a best practice model for indigenous primary healthcare services.

### As a precursor to a systematic review

Scoping reviews conducted as precursors to systematic reviews may enable authors to identify the nature of a broad field of evidence so that ensuing reviews can be assured of locating adequate numbers of relevant studies for inclusion. They also enable the relevant outcomes and target group or population for example for a particular intervention to be identified. This can have particular practical benefits for review teams undertaking reviews on less familiar topics and can assist the team to avoid undertaking an “empty” review [33]. Scoping reviews of this kind may help reviewers to develop and confirm their a priori inclusion criteria and ensure that the questions to be posed by their subsequent systematic review are able to be answered by available, relevant evidence. In this way, systematic reviews are able to be underpinned by a preliminary and evidence-based scoping stage.

A scoping review commissioned by the United Kingdom Department for International Development was undertaken to determine the scope and nature of literature on people’s experiences of microfinance. The results of this scoping review were used to inform the development of targeted systematic review questions that focussed upon areas of particular interest [34].

In their recent scoping review on the conduct and reporting of scoping reviews, Tricco and colleagues^10^ reveal only 12% of scoping reviews contained recommendations for the development of ensuing systematic reviews, suggesting that the majority of scoping review authors do not conduct scoping reviews as a precursor to future systematic reviews.

### To identify and analyze gaps in the knowledge base

Scoping reviews are rarely solely conducted to simply identify and analyze gaps present in a given knowledge base, as examination and presentation of what hasn’t been investigated or reported generally requires exhaustive examination of all of what is available. In any case, because scoping reviews tend to be a useful approach for reviewing evidence rapidly in emerging fields or topics, identification and analysis of knowledge gaps is a common and valuable indication for conducting a scoping review. A scoping review was recently conducted to review current research and identify knowledge gaps on the topic of “occupational balance”, or the balance of work, rest, sleep, and play [35]. Following a systematic search across a range of relevant databases, included studies were selected and in line with predetermined inclusion criteria, were described and mapped to provide both an overall picture of the current state of the evidence in the field and to identify and highlight knowledge gaps in the area. The results of the scoping review allowed the authors to illustrate several research ‘gaps’, including the absence of studies conducted outside of western societies, the lack of knowledge around peoples’ levels of occupational balance, as well as a dearth of evidence regarding how occupational balance may be enhanced. As with other scoping reviews focussed upon identifying and analyzing knowledge gaps, results such as these allow for the identification of future research initiatives.

## Discussion

Scoping reviews are now seen as a valid review approach for certain indications. A key difference between scoping reviews and systematic reviews is that in terms of a review question, a scoping review will have a broader “scope” than traditional systematic reviews with correspondingly more expansive inclusion criteria. In addition, scoping reviews differ from systematic reviews in their overriding purpose. We have previously recommended the use of the PCC mnemonic (Population, Concept and Context) to guide question development [36]. The importance of clearly defining the key questions and objectives of a scoping review has been discussed previously by one of the authors, as a lack of clarity can result in difficulties encountered later on in the review process [36].

Considering their differences from systematic reviews, scoping reviews should still not be confused with traditional literature reviews. Traditional literature reviews have been used as a means to summarise various publications or research on a particular topic for many years. In these traditional reviews, authors examine research reports in addition to conceptual or theoretical literature that focuses on the history, importance, and collective thinking around a topic, issue or concept. These types of reviews can be considered subjective, due to their substantial reliance on the author’s pre-exiting knowledge and experience and as they do not normally present an unbiased, exhaustive and systematic summary of a topic [12]. Regardless of some of these limitations, traditional literature reviews may still have some use in terms of providing an overview of a topic or issue. Scoping reviews provide a useful alternative to literature reviews when clarification around a concept or theory is required. If traditional literature reviews are contrasted with scoping reviews, the latter [6]:

- Are informed by an a priori protocol
- Are systematic and often include exhaustive searching for information
- Aim to be transparent and reproducible
- Include steps to reduce error and increase reliability (such as the inclusion of multiple reviewers)
- Ensure data is extracted and presented in a structured way

Another approach to evidence synthesis that has emerged recently is the production of evidence maps [37]. The purpose of these evidence maps is similar to scoping reviews to identify and analyse gaps in the knowledge base [37, 38]. In fact, most evidence mapping articles cite seminal scoping review guidance for their methods [38]. The two approaches therefore have many similarities, with perhaps the most prominent difference being the production of a visual database or schematic (i.e. map) which assists the user in interpreting where evidence exists and where there are gaps [38]. As Miake-Lye states, at this stage ‘it is difficult to determine where one method ends and the other begins.’ [38] Both approaches may be valid when the indication is for determining the extent of evidence on a particular topic, particularly when highlighting gaps in the research.

A further popular method to define and scope concepts, particularly in nursing, is through the conduct of a concept analysis [39–42]. Formal concept analysis is ‘a process whereby concepts are logically and systematically investigated to form clear and rigorously constructed conceptual definitions,’ [42] which is similar to scoping reviews where the indication is to clarify concepts in the literature. There is limited methodological guidance on how to conduct a concept analysis and recently they have been critiqued for having no impact on practice [39]. In our opinion, scoping reviews (where the purpose is to systematically investigate a concept in the literature) offer a methodologically rigorous alternative to concept analysis with their results perhaps being more useful to inform practice.

Comparing and contrasting the characteristics of traditional literature reviews, scoping reviews and systematic reviews may help clarify the true essence of these different types of reviews (see Table 1).

**Table 1:** Defining characteristics of traditional literature reviews, scoping reviews and systematic reviews

	Traditional Literature Reviews	Scoping reviews	Systematic reviews
A priori review protocol	No	Yes (some)	Yes
PROSPERO registration of the review protocol	No	No^a^	Yes
Explicit, transparent, peer reviewed search strategy	No	Yes	Yes
Standardized data extraction forms	No	Yes	Yes
Mandatory Critical Appraisal (Risk of Bias Assessment)	No	No^b^	Yes
Synthesis of findings from individual studies and the generation of ‘summary’ findings^c^	No	No	Yes

^a^Current situation; this may change in time. ^b^Critical appraisal is not mandatory, however, reviewers may decide to assess and report the risk of bias in scoping reviews. ^c^By using statistical meta-analysis (for quantitative effectiveness, or prevalence or incidence, diagnostic accuracy, aetiology or risk, prognostic or psychometric data), or meta-synthesis (experiential or expert opinion data) or both in mixed methods reviews

Rapid reviews are another emerging type of evidence synthesis and a substantial amount of literature have addressed these types of reviews [43–47]. There are various definitions for rapid reviews, and for simplification purposes, we define these review types as ‘systematic reviews with shortcuts.’ In this paper, we have not discussed the choice between a rapid or systematic review approach as we are of the opinion that perhaps the major consideration for conducting a rapid review (as compared to a systematic or scoping review) is not the purpose/question itself, but the feasibility of conducting a full review given financial/resource limitations and time pressures. As such, a rapid review could potentially be conducted for any of the indications listed above for the scoping or systematic review, whilst shortening or skipping entirely some steps in the standard systematic or scoping review process.

There is some overlap across the six listed purposes for conducting a scoping review described in this paper. For example, it is logical to presume that if a review group were aiming to identify the types of available evidence in a field they would also be interested in identifying and analysing gaps in the knowledge base. Other combinations of purposes for scoping reviews would also make sense for certain questions/aims. However, we have chosen to list them as discrete reasons in this paper in an effort to provide some much needed clarity on the appropriate purposes for conducting scoping reviews. As such, scoping review authors should not interpret our list of indications as a discrete list where only one purpose can be identified.

It is important to mention some potential abuses of scoping reviews. Reviewers may conduct a scoping review as an alternative to a systematic review in order to avoid the critical appraisal stage of the review and expedite the process, thinking that a scoping review may be easier than a systematic review to conduct. Other reviewers may conduct a scoping review in order to ‘map’ the literature when there is no obvious need for ‘mapping’ in this particular subject area. Others may conduct a scoping review with very broad questions as an alternative to investing the time and effort required to craft the necessary specific questions required for undertaking a systematic review. In these cases, scoping reviews are not appropriate and authors should refer to our guidance regarding whether they should be conducting a systematic review instead.

This article provides some clarification on when to conduct a scoping review as compared to a systematic review and clear guidance on the purposes for conducting a scoping review. We hope that this paper will provide a useful addition to this evolving methodology and encourage others to review, modify and build upon these indications as the approach matures. Further work in scoping review methods is required, with perhaps the most important advancement being the recent development of an extension to the Preferred Reporting Items for Systematic Reviews and Meta-Analyses (PRISMA) for scoping reviews [48] and the development of software and training programs to support these reviews [49, 50]. As the methodology advances, guidance for scoping reviews (such as that included in the Joanna Briggs Institute Reviewer’s Manual) will require revision, refining and updating.

## Conclusion

Scoping reviews are a useful tool in the ever increasing arsenal of evidence synthesis approaches. Researchers may preference the conduct of a scoping review over a systematic review where the purpose of the review is to identify knowledge gaps, scope a body of literature, clarify concepts, investigate research conduct, or to inform a systematic review. Although conducted for different purposes compared to systematic reviews, scoping reviews still require rigorous and transparent methods in their conduct to ensure that the results are trustworthy. Our hope is that with clear guidance available regarding whether to conduct a scoping review or a systematic review, there will be less scoping reviews being performed for inappropriate indications better served by a systematic review, and vice-versa.

## References

[1] Bastian H, Glasziou P, Chalmers I (2010). Seventy-five trials and eleven systematic reviews a day: how will we ever keep up?. PLoS Med.

[2] Chalmers I, Hedges LV, Cooper H (2002). A brief history of research synthesis. Eval Health Prof.

[3] Jordan Z, Munn Z, Aromataris E, Lockwood C (2015). Now that we're here, where are we? The JBI approach to evidence-based healthcare 20 years on. Int J Evid Based Healthc..

[4] Munn Z, Stern C, Aromataris E, Lockwood C, Jordan Z (2018). What kind of systematic review should I conduct? A proposed typology and guidance for systematic reviewers in the medical and health sciences. BMC Med Res Methodol.

[5] Arksey H, O'Malley L (2005). Scoping studies: towards a methodological framework. Int J Soc Res Methodol.

[6] Peters MD, Godfrey CM, Khalil H, McInerney P, Parker D, Soares CB (2015). Guidance for conducting systematic scoping reviews. Int J Evid Based Healthc.

[7] Levac D, Colquhoun H, O'Brien KK (2010). Scoping studies: advancing the methodology. Implement Sci.

[8] Colquhoun HL, Levac D, O'Brien KK (2014). Scoping reviews: time for clarity in definition, methods, and reporting. J Clin Epidemiol.

[9] Pham MT, Rajić A, Greig JD, Sargeant JM, Papadopoulos A, McEwen SA (2014). A scoping review of scoping reviews: advancing the approach and enhancing the consistency. Res Synth Methods.

[10] Tricco AC, Lillie E, Zarin W (2016). A scoping review on the conduct and reporting of scoping reviews. BMC Med Res Methodol.

[11] Pearson A (2004). Balancing the evidence: incorporating the synthesis of qualitative data into systematic reviews. JBI Reports.

[12] Aromataris E, Pearson A (2014). The systematic review: an overview. AJN The American Journal of Nursing.

[13] Liberati A, Altman DG, Tetzlaff J (2009). The PRISMA statement for reporting systematic reviews and meta-analyses of studies that evaluate healthcare interventions: explanation and elaboration. BMJ (Clinical research ed).

[14] Higgins J, Green S, eds. Cochrane handbook for systematic reviews of interventions. Version 5.1.0 [updated March 2011]. ed: The Cochrane Collaboration 2011.

[15] Munn Z, Porritt K, Lockwood C, Aromataris E, Pearson A (2014). Establishing confidence in the output of qualitative research synthesis: the ConQual approach. BMC Med Res Methodol.

[16] Pearson A, Jordan Z, Munn Z (2012). Translational science and evidence-based healthcare: a clarification and reconceptualization of how knowledge is generated and used in healthcare. Nursing research and practice.

[17] Steinberg E, Greenfield S, Mancher M, Wolman DM, Graham R. Clinical practice guidelines we can trust. Institute of Medicine. Washington, DC: National Academies Press; 2011. 24983061

[18] Gough D, Thomas J, Oliver S (2012). Clarifying differences between review designs and methods. Systematic Reviews.

[19] Grant MJ, Booth A (2009). A typology of reviews: an analysis of 14 review types and associated methodologies. Health Inf Libr J.

[20] Tricco AC, Tetzlaff J, Moher D (2011). The art and science of knowledge synthesis. J Clin Epidemiol.

[21] Armstrong R, Hall BJ, Doyle J, Waters E (2011). ‘Scoping the scope’ of a cochrane review. J Public Health.

[22] Anderson S, Allen P, Peckham S, Goodwin N (2008). Asking the right questions: scoping studies in the commissioning of research on the organisation and delivery of health services. Health Research Policy and Systems.

[23] Pearson A, Wiechula R, Court A, Lockwood C (2005). The JBI model of evidence-based healthcare. International Journal of Evidence-Based Healthcare.

[24] Bragge P, Clavisi O, Turner T, Tavender E, Collie A, Gruen RL (2011). The global evidence mapping initiative: scoping research in broad topic areas. BMC Med Res Methodol.

[25] Chambers D, Wilson PM, Thompson CA, Hanbury A, Farley K, Light K (2011). Maximizing the impact of systematic reviews in health care decision making: a systematic scoping review of knowledge-translation resources. Milbank Q.

[26] Challen K, Lee AC, Booth A, Gardois P, Woods HB, Goodacre SW (2012). Where is the evidence for emergency planning: a scoping review. BMC Public Health.

[27] Schaink AK, Kuluski K, Lyons RF (2012). A scoping review and thematic classification of patient complexity: offering a unifying framework. Journal of comorbidity.

[28] Hines Delaney, Modi Neena, Lee Shoo K., Isayama Tetsuya, Sjörs Gunnar, Gagliardi Luigi, Lehtonen Liisa, Vento Maximo, Kusuda Satoshi, Bassler Dirk, Mori Rintaro, Reichman Brian, Håkansson Stellan, Darlow Brian A., Adams Mark, Rusconi Franca, San Feliciano Laura, Lui Kei, Morisaki Naho, Musrap Natasha, Shah Prakesh S. (2016). Scoping review shows wide variation in the definitions of bronchopulmonary dysplasia in preterm infants and calls for a consensus. Acta Paediatrica.

[29] Callary SA, Solomon LB, Holubowycz OT, Campbell DG, Munn Z, Howie DW (2015). Wear of highly crosslinked polyethylene acetabular components. Acta Orthop.

[30] Davy C, Harfield S, McArthur A, Munn Z, Brown A (2016). Access to primary health care services for indigenous peoples: a framework synthesis. Int J Equity Health.

[31] Harfield S, Davy C, Kite E (2015). Characteristics of indigenous primary health care models of service delivery: a scoping review protocol. JBI Database System Rev Implement Rep.

[32] Harfield SG, Davy C, McArthur A, Munn Z, Brown A, Brown N (2018). Characteristics of indigenous primary health care service delivery models: a systematic scoping review. Glob Health.

[33] Peters MDJ LC, Munn Z, Moola S, Mishra RK (2015) , Protocol. Adelaide: the Joanna Briggs Institute UoA. What are people’s views and experiences of delivering and participating in microfinance interventions? A systematic review of qualitative evidence from South Asia.

[34] Peters MDJ LC, Munn Z, Moola S, Mishra RK People’s views and experiences of participating in microfinance interventions: A systematic review of qualitative evidence. London: EPPI-Centre: social science research unit, UCL Institute of education, University College London; 2016.

[35] Wagman P, Håkansson C, Jonsson H (2015). Occupational balance: a scoping review of current research and identified knowledge gaps. J Occup Sci.

[36] Peters MD (2016). In no uncertain terms: the importance of a defined objective in scoping reviews. JBI Database System Rev Implement Rep.

[37] Hetrick SE, Parker AG, Callahan P, Purcell R (2010). Evidence mapping: illustrating an emerging methodology to improve evidence-based practice in youth mental health. J Eval Clin Pract.

[38] Miake-Lye IM, Hempel S, Shanman R, Shekelle PG (2016). What is an evidence map? A systematic review of published evidence maps and their definitions, methods, and products. Systematic reviews.

[39] Draper P (2014). A critique of concept analysis. J Adv Nurs.

[40] Gibson CH (1991). A concept analysis of empowerment. J Adv Nurs.

[41] Meeberg GA (1993). Quality of life: a concept analysis. J Adv Nurs.

[42] Ream E, Richardson A (1996). Fatigue: a concept analysis. Int J Nurs Stud.

[43] Tricco AC, Antony J, Zarin W (2015). A scoping review of rapid review methods. BMC Med.

[44] Ganann R, Ciliska D, Thomas H (2010). Expediting systematic reviews: methods and implications of rapid reviews. Implement Sci.

[45] Harker J, Kleijnen J (2012). What is a rapid review? A methodological exploration of rapid reviews in health technology assessments. Int J Evid Based Healthc.

[46] Khangura S, Konnyu K, Cushman R, Grimshaw J, Moher D (2012). Evidence summaries: the evolution of a rapid review approach. Syst Rev.

[47] Munn Z, Lockwood C, Moola S (2015). The development and use of evidence summaries for point of care information systems: a streamlined rapid review approach. Worldviews Evid-Based Nurs.

[48] Tricco AC, Lillie E, Zarin W (2018). PRISMA extension for scoping reviews (PRISMA-ScR): checklist and explanation. Ann Intern Med.

[49] Munn Z, Aromataris E, Tufanaru C, Stern C, Porritt K, Farrow J, Lockwood C, Stephenson M, Moola S, Lizarondo L, McArthur A. The development of software to support multiple systematic review types: the Joanna Briggs institute system for the unified management, assessment and review of information (JBI SUMARI). Int J Evid Based Healthc. 2018. (in press) 10.1097/XEB.0000000000000152 30239357

[50] Stern C, Munn Z, Porritt K (2018). An international educational training course for conducting systematic reviews in health care: the Joanna Briggs Institute's comprehensive systematic review training program. Worldviews Evid-Based Nurs.

